# Effect of Replacing Alfalfa Hay with Common Vetch Hay in Sheep Diets on Growth Performance, Rumen Fermentation and Rumen Microbiota

**DOI:** 10.3390/ani14152182

**Published:** 2024-07-26

**Authors:** Chunhuan Ren, Xiaoan Zhang, Huiqing Wei, Sunze Wang, Wenjie Wang, Li He, Yuan Lu, Kefan Zhang, Zijun Zhang, Guanjun Wang, Yafeng Huang

**Affiliations:** 1 College of Animal Science and Technology, Anhui Agricultural University, Hefei 230036, China; renchunhuan@ahau.edu.cn (C.R.); zhangxiaoan@stu.ahau.edu.cn (X.Z.); weihuiqing123@stu.ahau.edu.cn (H.W.); wangsunze@stu.ahau.edu.cn (S.W.); wangwenjie0123@stu.ahau.edu.cn (W.W.); luyuan6868@stu.ahau.edu.cn (Y.L.); zhangkefan@stu.ahau.edu.cn (K.Z.); zhangzijun@ahau.edu.cn (Z.Z.); 2 National Agricultural Green Development Long-Term Fixed Observation Yingshang Test Station, Fuyang 236200, China; 3 Center of Agriculture Technology Cooperation and Promotion of Dingyuan County, Chuzhou 233200, China; 4 New Rural Development Research Institute, Anhui Agricultural University, Hefei 230036, China; heli@ahau.edu.cn

**Keywords:** common vetch, growth performance, rumen fermentation, microbiota composition, sheep

## Abstract

**Simple Summary:**

Using homegrown forage instead of imported alfalfa hay (AH) improves animal productivity and reduces feed costs, but the benefits of feeding high-quality common vetch hay (CVH) to lambs are not clear. This study aimed to determine the effect of replacing alfalfa hay with common vetch hay on growth performance, rumen fermentation, and rumen microbiota of fattening lambs. The results showed that substitution of AH by CVH in ruminant diets result in improvements in fattening performance and economic efficiency and a reduction in the methane produced per unit of ADG, thereby resulting in advantageous in decreasing reliance on imported AH.

**Abstract:**

The aim of this study was to determine whether the inclusion of 40% of common vetch (CV) hay as a feed ingredient in place of alfalfa hay (AH) would improve performance and ruminal fermentation and microbiota in fattening lambs. Twenty lambs were equally divided into two groups: control group (fed 40% AH with 20% rice straw) and CV group (fed 40% CV hay with 20% rice straw). Concerning hay quality, CV hay had greater in vitro digestibility of dry matter and neutral detergent fiber (*p* < 0.05) than AH. Lambs fed the CV diet had a higher average daily gain (ADG) and efficiency of feed and economy than lambs fed the control group. The NH_3_-N content and estimated methane produced per unit of ADG of the CV diet group were significantly lower (*p* < 0.05) than control group. Multiple differential microbial genera were identified, with *Prevotella* being the most dominant genus and a tendency towards higher (*p* = 0.095) in lambs offered CV diet. The higher *Ruminococcus* abundance (*p* < 0.05) was found in animals of the CV group compared to the control group. In summary, CV can be incorporated into lamb diets as a low-cost forage alternative to AH to improve feed efficiency and animal performance and to reduce methane produced per unit of ADG.

## 1. Introduction

As the largest lamb meat producer worldwide, China had 194.03 million sheep and 132.24 million goats as of 2022, producing 5.24 million tons of mutton and goat meat and 14,648.53 tons of cashmere wool [1,2]. However, the large-scale confined feeding of high-energy grains to sheep in China and worldwide is faced with increasing challenges in terms of animal health and welfare, lack of adequate and high-quality forage, and high feed prices [3,4]. According to the data of the General Administration of Customs of the People’s Republic of China, China imported 1.94 million tons of dried forages hay, containing 1.78 million tons of alfalfa (*Medicago sativa* L.) hay (AH) [5]. Hence, improvement of forage self-sufficiency to replace use of AH can be known as insurgent for the sheep industry for farmers, particularly at times of protein feed shortage during the winter and spring seasons.

Common vetch (CV) is one of the most widely distributed annual leguminous crops throughout the northern hemisphere, originating in the arid regions of the Middle East [6,7]. As a cool-season annual legume, CV is mainly utilized as an alternative high-protein feed for ruminants and as green manure. Its yield is somewhat high, even under the extreme winter conditions and on the Tibetan plateau, because of its high adaptability and cold resistance [6,8]. In a previous study, replacing AH with CV hay resulted in similar performance and lower methane (CH_4_) emissions in growing cattle [9]. Moreover, Huang et al. [6] concluded that a diet containing 20% common vetch hay improved growth performance and the digestibility of dietary nutrients of growing lambs compared to a diet of 20% AH. Meanwhile, rice (*Oryza sativa* L.) straw (RS) is one of the most abundant crop residues in China, with nearly 21 million metric tons produced annually [10]. However, RS is often considered waste that is discarded or burnt, leading to wasted resources and directly generating massive pollution as dust and greenhouse gases [11].

In ruminants, rumen microorganisms can use their unique physiological structure to convert the roughage into volatile fatty acids and proteins, providing sufficient nutrients for the animals [12,13]. Notably, the stability of the rumen microflora has a positive effect on the host’s health and immune system and maintains performance and product quality [14,15]. Similarly, the composition and structure of the diet strongly affect the composition of rumen microbiota [16,17,18]. Zhang et al. [4] also confirmed that feeding cattle high-quality forage promotes healthy and appropriate changes in the rumen microbiome in the long term. Methane emissions from ruminant livestock have attracted significant attention in many countries of the worldwide, particularly in China. This is mainly due to global CH_4_ production from ruminants accounting for 23–27% of global anthropogenic CH_4_ emissions to cause warming phenomenon [19]. Previous studies have reported that CH_4_ emission varied with the source of forages used in the ruminant diet [20,21], suggesting that diet affects methane output.

Considering the importance of the sheep industry, forage utilization, and the continuous search for efficient feeding strategies, this study compared the growth performance, ruminal fermentation, and microbiota and CH_4_ emission of lambs offered CV hay or AH combined with rice straw. The aim of this study was to evaluate the effect of replacing AH with CV hay on growth performance, rumen fermentation, predicted CH_4_ emission, and rumen microbiota of fattening lambs. Results revealed the potential nutritional value of CV hay and provided a theoretical basis and practical example of increased efficiency and sustainable development of the sheep industry.

## 2. Materials and Methods

### 2.1. Study Site, Common Vetch Harvest, and Hay Production

Common vetch (Lanjian No.2) was sown at a density of 150 seeds/m^2^ in rows spaced 20 cm apart in fields at the National Agricultural Green Development Long-Term Fixed Observation Yingshang Test Station (at Yingshang City, Anhui Province, China: 32°42′18″ N, 116°0′13″ E; altitude 20.7 m). The study site has a transitional climate between the north temperate and subtropical region, with an average annual temperature of 15.0 °C, a frost-free period of 221 days, and an average annual rainfall of 904.6 mm. The CV was sown in late October 2022, manually harvested during the flowering stage in mid-April, and subsequently air-dried for four days before being stored for the feeding experiment.

### 2.2. Study Animals, Experimental Design, and Growth Performance

The feed trial was conducted from early August 2023 to late September 2023 in Yingshang County, Anhui Province, China. A total of 20 Huang-huai male lambs with an initial live weight (BW) of 24.89 ± 0.67 kg at four months of age were randomly divided into two groups of 10 and housed in separate pens (3.1 × 1.5 m) with two animals in each pen. The control group was fed total mixed ration (TMR) pellets consisting of 400 g/kg concentrate mixtures, 400 g/kg AH (as fed, CON), and 200 g/kg brittle culm RS. The other group received similar feed with the AH substituted with CV hay (as fed, CVG). The diets were prepared based on the nutritional requirements of meat-type sheep and goats with the crude protein (CP) and metabolizable energy (ME) totaling 14.4% DM and 13.0 KJ/g DM, respectively (NY/T 816-2021, Ministry of Agriculture, China) [22]. Then, full-price pellet feed was processed; its composition and nutritional value are shown in Table 1.

All animals received treatment against internal and external parasites using an anthelmintic drug before the experiment. The experiment lasted 64 days, including a 14-day adaptation period and a 50-day fattening period, during which growth performance was measured and ruminal fluids were collected. All lambs received water ad libitum and were fed twice daily at 08:30 and 18:30. Two-day averages of body weight (BW) of lambs were recorded at the beginning and end of the fattening period before the morning feed. The amount of feed dispensed to each lamb was recorded daily and the refusals were weighed every three days. The average daily feed intake (ADFI), average daily gain (ADG), and feed conversion ratio (FCR) for the whole period were calculated as described by Huang et al. [6].

### 2.3. Ruminal Fluid Collection and Fermentation Analysis

Ruminal fluid samples (approximately 30 mL) were collected from six selected lambs per group on day 65 after overnight fasting using an oral gastric tube (MDW15, Colebo Equipment Co., Ltd., Wuhan, China) and a 350-mL syringe. The pH values of rumen fluid samples were measured immediately after collection using a portable pH meter (PHS-3C, Leici Scientific Instrument Co., Ltd., Shanghai, China). Each sample of ruminal fluid was strained through four layers of cheesecloth and divided into three parts. One 5 mL strained ruminal fluid sample was mixed with 1 mL of 25% metaphosphoric acid and stored at −20 °C until it was analyzed for volatile fatty acids (VFA). The rest of the ruminal fluid was immediately frozen in liquid nitrogen and stored at −80 °C until analysis of NH_3_-N and DNA extraction. The VFA analysis was conducted using capillary gas chromatograph (C-2010 Plus, Shimadzu, Japan) equipped with a capillary column (30 mm × 0.32 mm × 0.25 mm) following the method described by Huang et al. [6]. Ruminal NH_3_-N content was measured based on a colorimetric method [24] and adapted for Thermo Scientific Multiskan GO (BIO-RAD, Hercules, CA, USA) with a 700 nm absorbance filter.

### 2.4. Ruminal Fluid DNA Extraction, Microbial Sequencing and Analysis

To determine the ruminal fluid microbial composition, high-throughput sequencing was performed at Majorbio Bio-Pharm Technology Co., Ltd. (Shanghai, China). The total DNA in the ruminal fluid was extracted using the QIAamp DNA Stool Mini Kit (Qiagen, Shanghai, China) according to the manufacturer’s instructions. DNA purity and quality assessed using electrophoresis (2% agarose gel) and an OD-1000+ spectrophotometer (one drop, Shanghai, China). The V3-V4 regions of the bacterial 16S rRNA gene were amplified using primers 338F (ACTCCTACGGGGGCAGCA) and 806R (GGACTACHVGGG TWTCTAAT) [25]. Deep sequencing of DNA extracts was conducted on an Illumina MiSeq platform (Illumina, San Diego, CA, USA; Illumina Novaseq6000) using paired-end sequencing (2 × 250 bp).

The raw data were initially processed and analyzed by Vsearch (v2.13.4 linux x86 64) software. Operational taxonomic units (OTUs) were clustered at a similarity level of 97% using the UPARSE software (version 7.1) [26], and chimeric sequences were identified and removed. The AS Venn analysis was performed on the OTUs obtained. Representative sequences of OTUs were aligned to the SILVA database for taxonomic assignments of bacteria using QIIME (http://qiime.org/scripts/assign_taxonomy.html accessed on 19 March 2024) [27]. The observed OTUs and the Shannon index were used to assess the alpha diversity of each rumen fluid sample. Mann–Whitney U tests and Wilcoxon signed-rank tests were used to analyze the variance in the microbial data.

Richness estimates and diversity indices, including Chao 1, Good’s coverage, Shannon index, and Simpson’s index, were calculated using the QIIME Pipeline (Version 1.8.0). A principal coordinate analysis (PCoA) based on unweighted UniFrac distances was conducted to compare all samples, and a distance-based matrix analysis (PERMANOVA) was performed to evaluate differences between samples using the vegan package in the QIIME2 (2019.4) software.

### 2.5. In Vitro Digestibility

Samples of CV hay and AH were oven-dried at 65 °C for 48 h and ground through a 1 mm sieve for in vitro digestibility determination. Then, samples of CV hay and AH were analyzed to determine the in vitro digestibility of dry matter (IVDMD) and neutral detergent fiber (IVNDFD) using an artificial rumen incubator (MC-NFSF-II, Beijing Mancang Technology, Beijing, China). Ruminal fluid was collected 3 min postmortem from two slaughtered Huang-huai sheep rams fed a TMR of 40% CV, 20.0% RS, and 40.0% concentrate. The collected ruminal fluid was strained through four layers of sterilized cheesecloth and intermittently stirred under CO_2_ flushing in a water bath (39 °C). Ground samples (250 mg/bag, in duplicate) were weighted into pre-weighed filter bags (5.2 × 4.7 cm, 30 μm pore size) and then heat-sealed. Then, twenty-four bags plus three blank controls were incubated in each incubation jar. Incubation jars were filled with 1596 mL pre-warmed (39 °C) mineral buffer and 400 mL ruminal fluid and incubated for 48 h at 39 °C with incubation. After the incubation, the bags were removed and gently washed with cold tap water, and the α-amylase-treated neutral detergent fiber (aNDF) content was analyzed. Residual NDF content was analyzed as described by Van Soest [28], with the addition of α-amylase and anhydrous sodium sulfite. These residual fractions were calculated as IVDMD (g/kg DM) and IVNDFD (g/kg NDF) using the equations recommended by Huang et al. [7].

### 2.6. Calculations and Statistical Analysis

Statistical analyses were performed using SPSS software (Version 25.0. IBM Corporation, Armonk, NY, USA). Data obtained for in vitro digestibility, growth performance, ruminal fermentation parameters, and predicted CH_4_ emission were analyzed by one-way analysis of variance. Differences between groups were evaluated using *t*-test with significance defined as *p* < 0.05, and a tendency was defined as 0.05 < *p* ≤ 0.10. Pearson’s correlation was used to evaluate the correlations among bacterial populations, ADG, rumen VFA and methane emission.

## 3. Results

### 3.1. Hay In Vitro Digestibility and Growth Performances

**Figure 1:**
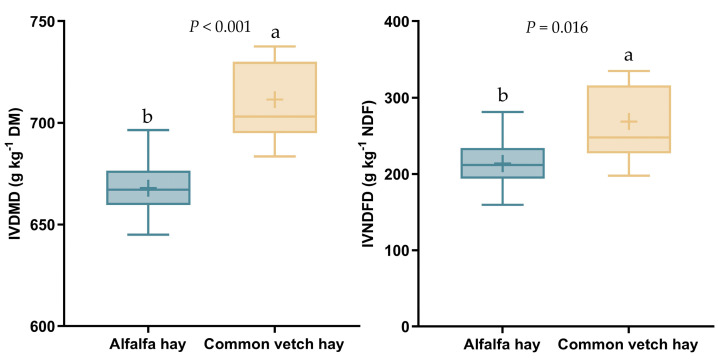
In vitro digestibility of the hay from common vetch and alfalfa. ^a,b^ Values within a row with different lowercase superscript letters differ significantly at *p* < 0.05; Abbreviations: IVDMD = in vitro dry matter digestibility; IVNDFD = in vitro neutral detergent fiber digestibility.

As shown in Figure 1, the IVDMD and IVNDFD concentrations of CV hay were higher (*p* < 0.05) than those of alfalfa hay.

**Figure 2:**
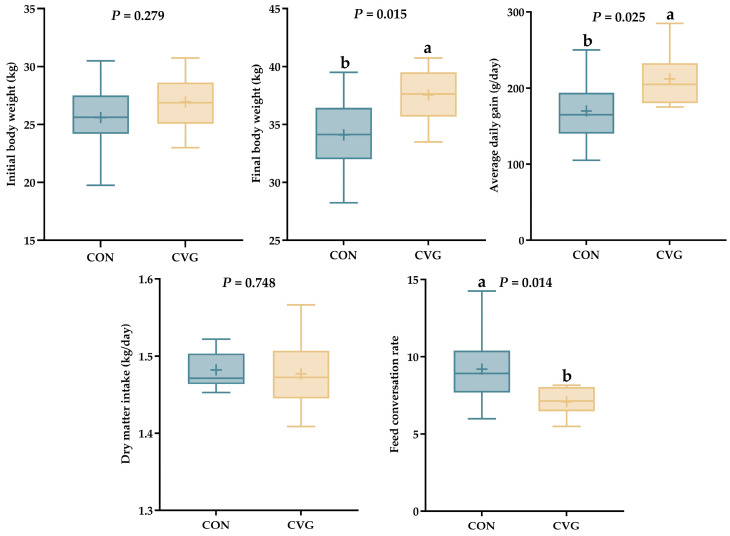
Effects of common vetch hay on growth performance and feed conversion rate of growing lambs. ^a,b^ Values within a row with different lowercase superscript letters differ significantly at *p* < 0.05; Abbreviations: CON = diet containing alfalfa hay; CVG = diet containing common vetch hay.

The initial BW of the lambs did not differ (*p* > 0.05) between the groups and averaged 26.28 kg (Figure 2), but the final BW was higher (*p* < 0.05) in the CVG group than in CON group. The ADG of lambs from the CVG group was markedly (*p* < 0.05) higher than that of lambs from the CON group. The AFI was not affected by the diet (*p* > 0.05), but FCR was 23.0% lower (*p* < 0.05) in the CVG group than in the CON group.

### 3.2. Ruminal Fermentation Characteristics

Ruminal pH was similar (*p* > 0.05) between the two groups and averaged 5.75 and 6.08 in the CON and CVG groups, respectively (Table 2). Ruminal NH_3_-N concentration was significantly lower (*p* < 0.05) in the CVG group compared with the CON group. The concentrations of total VFA were not affected between diet and averaged 58.91 mmol/L. Likewise, there was no significant difference (*p* > 0.05) in the molar proportion of propionate, isobutyrate, and acetate:propionate ratio between the two groups. However, the molar proportions of acetate and valerate were lower (*p* < 0.05) in the CVG group than in the CON group. Additionally, the molar proportions of butyrate and isovalerate were significantly higher (*p* < 0.05) in the CVG group as compared to the CON group.

### 3.3. Methane Emission

The predicted CH_4_ emissions are presented in Table 3. For per unit of AFI, the predicted CH_4_ values from lambs fed the CVG diet were similar to those from lambs fed the CON diet. Per unit of ADG, compared to the CON diet, lambs fed the CVG diet had a lesser predicted CH_4_ production (*p* < 0.05).

### 3.4. Ruminal Microbial Community Composition

#### 3.4.1. Alpha Diversity and Relative Abundance of Bacteria

**Figure 3:**
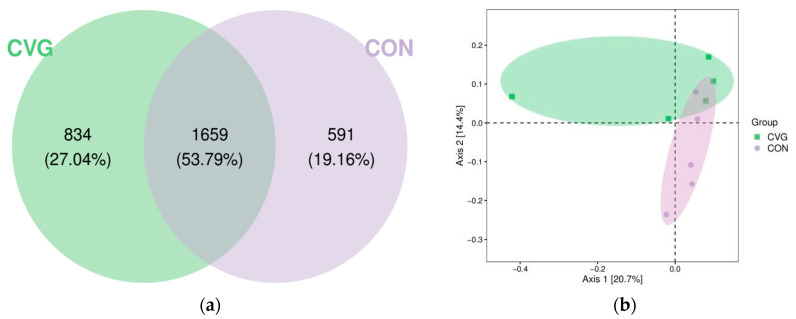
Venn analysis of operational taxonomic units (**a**) and principal coordinate analysis of the beta diversity (**b**) of the ruminal microbiota of sheep fed CON and CVG diets. CON = diet containing alfalfa hay; CVG = diet containing common vetch hay.

A total of 593,394 effective reads were retrieved from 10 sequenced samples after filtering out low-quality reads with an average of 59,339 per sample, and 3084 OTUs were identified with a similarity of 97%. Venn analysis identified 591 and 834 unique OTUs in the CON and CVG groups (Figure 3a), respectively.

Similarly, the Good’s coverage index for all samples exceeded 99%, implying that the sequencing depth was adequate for representing each rumen microbial community. After analysis using QIIME Pipeline, alpha diversity indices showed that there was no significant difference in Chao1, Simpson’s index, Shannon index, and Goods_coverage between the two treatment groups (Table 4). The PCoA based on unweighted UniFrac distances showed a relative separation of rumen bacteria in the CON and CVG groups (Figure 3b). The PCoA1 and PCoA2 could explain 20.7% and 14.4% of the total community, respectively.

**Figure 4:**
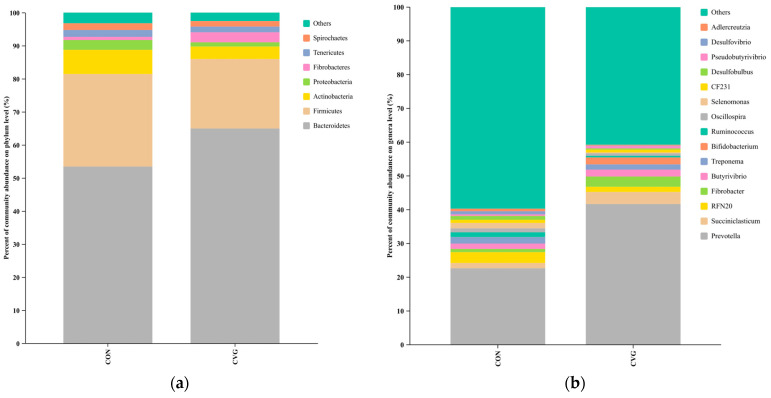
Relative abundance of rumen bacteria at the (**a**) phylum and (**b**) genus levels of sheep fed CON and CVG diets. CON = diet containing alfalfa hay; CVG = diet containing common vetch hay.

Taxonomic analysis classified the bacteria into 321 genera belonging to 24 phyla. At the phylum level, seven bacterial phyla were identified to as the detected bacterial phyla (relative abundance > 1%; Figure 4a). The most abundant phyla in the CON and CVG groups were *Bacteroidetes* (53.42% and 64.95%), followed by *Firmicutes* (28.00% and 21.01%), *Actinobacteria* (7.31% and 3.77%), *Proteobacteria* (2.96% and 1.29%), *Fibrobacteres* (0.96% and 3.03%), *Tenericutes* (2.08% and 2.08%), and *Spirochaetes* (2.01% and 1.64%), respectively. The relative abundances of seven phyla showed no significant differences between the two treatment groups, but there was a tendency for higher *Proteobacteria* in CVG group compared to the CON group (*p* = 0.076; Supplementary Table S1). At the genus level, the top 15 genera were identified and the most abundant genus in CON and CVG groups were *Prevotella* (22.65% and 41.67%) (Figure 4b; Supplementary Table S2). *Ruminococcus* abundance was higher in the CON group than in the CVG group (*p* < 0.05), while abundance of *Prevotella* and *Succiniclasticum* tended to be higher (*p* = 0.095) and *Desulfobulbus* and *Adlercreutzia* tended to be lower (*p* = 0.095) in the CVG group than in the CON group.

**Figure 5:**
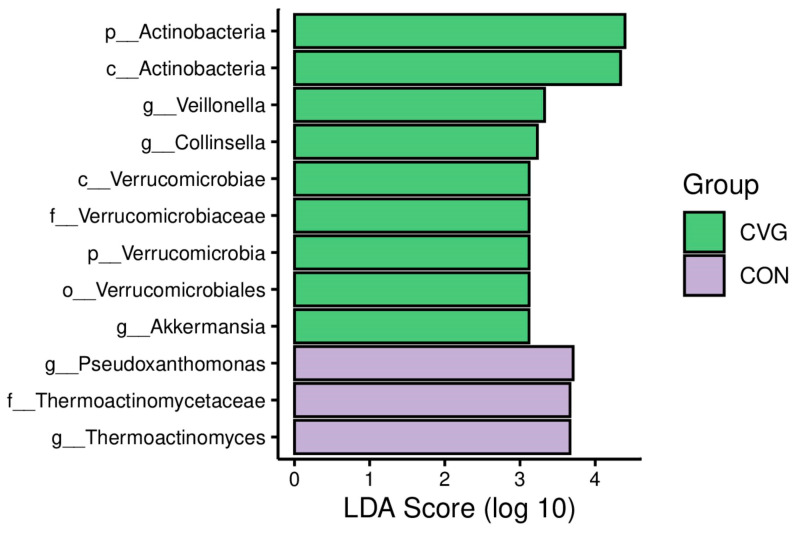
The linear discriminate analysis threshold of 3.0 at the genus level of the ruminal microbiota of sheep fed CON and CVG diets. CON = diet containing alfalfa hay; CVG = diet containing common vetch hay.

The LEfSe analysis identified 12 core genera with an LDA score > 3.0. *Pseudoxanthomonas* and *Thermoactinomyces* were annotated at the genera level and were found to be enriched in the CON group. However, genera such as *Veillonella*, *Collinsella*, and *Akkermansia* were enriched in the CVG group (Figure 5).

#### 3.4.2. Correlation Analysis

**Figure 6:**
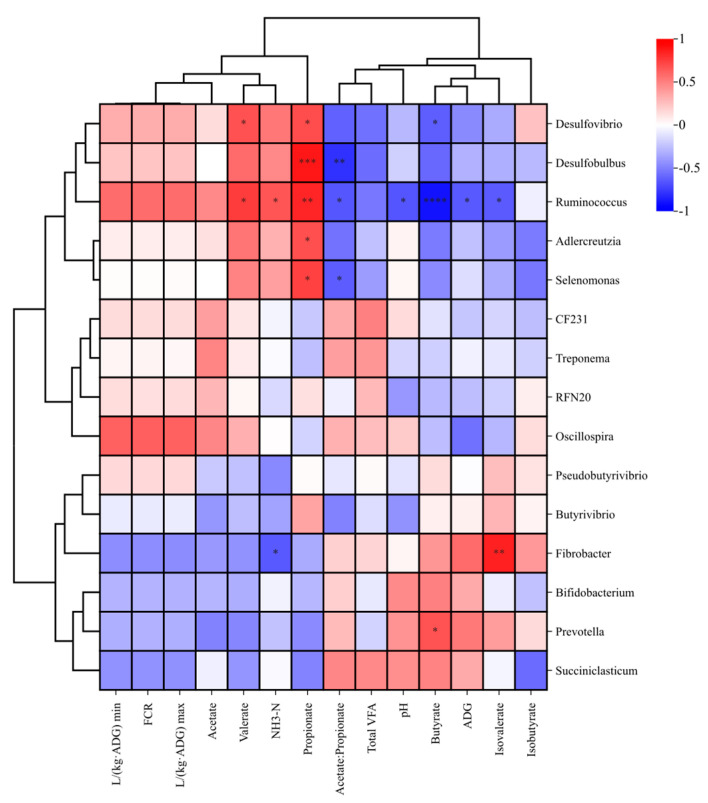
Heat map summarizing the Pearson’s correlations between different ruminal microbiomes and ADG, rumen VFA, and methane emission. The color intensity and circle size are proportional to the correlation values; * 0.01 < *p* ≤ 0.05; ** 0.001 < *p* ≤ 0.01; *** *p* < 0.001; **** *p* < 0.0001. ADG, average daily gain; FCR, feed conversion ratio; VFA, volatile fatty acids.

Pearson’s correlation analysis was performed to explore the relationships between ruminal microbiota and ADG, rumen VFA, and methane emission (Figure 6). The ADG and pH was strongly negatively correlated (*p* < 0.05) with the genera *Ruminococcus*. Ruminal NH_3_-N was negatively correlated (*p* < 0.05) with *Fibrobacter* but positively correlated (*p* < 0.05) with *Ruminococcus.* Ruminal valerate was positively correlated (*p* < 0.05) with *Ruminococcus* and *Desulfovibrio*, while ruminal acetate: propionate was negatively correlated (*p* < 0.05) with *Ruminococcus*, *Selenomonas* and *Desulfobulbus.* Ruminal propionate was positively correlated (*p* < 0.05) with *Ruminococcus*, *Selenomonas*, *Desulfobulbus*, *Desulfovibrio*, and *Adlercreutzia*, whereas ruminal butyrate was negatively correlated (*p* < 0.05) with *Ruminococcus* and *Desulfovibrio*. Ruminal isovalerate was negatively correlated (*p* < 0.05) with *Ruminococcus* but positively correlated (*p* < 0.05) with *Fibrobacter.*

## 4. Discussion

Limited research is available on the effects of CV hay in combination with RS fed to feedlot lambs; therefore, this study will assist the intensification of cultivated CV hay-based livestock systems to enhance forages sourced for animal production. The present study showed that CV hay affected the growth performance, ruminal VFA formation and bacteria, and methane emission of growing lambs.

### 4.1. Hay In Vitro Digestibility and Growth Performances

Forage quality is key in the evaluation of animal performance and the production potential of a forage crop [21]. The greater IVDMD and IVNDFD of the CV hay compared to the AH suggest that CV hay allows greater nutrient utilization efficiency in lambs and may improve the growth performance of lambs. Similarly, Huang et al. [6] reported that replacing AH (20% of the diet, as fed) with CV hay in the diet of fattening lambs resulted in greater apparent total tract digestibility of NDF.

Providing a sufficient diet for growing lambs is critical to ensure their production and health. The AFI of lambs did not differ between CV hay and AH, indicating that CV hay was just as palatable as AH. The CV hay supported high ADG in lambs, indicating that it is a high-quality forage substitute for AH and promotes growth performance. The difference in daily body mass gain between the two treatment groups appeared to be caused by differences in the digestibility of nutrients since there was no difference in DMI. These results were consistent with Huang et al. [6], who reported that replacing AH (20% of the diet, as fed) with CV hay for fattening lambs resulted in greater body mass gain and similar DMI.

Overall, our findings indicate that the feeding value of CV hay prepared at the full-bloom stage is superior to that of AH, based on the higher growth performance and the lower feed conversion ratio of lambs fed CV diets. This is a meaningful example of the cost-efficiency of the use of CV hay in feedlots.

### 4.2. Ruminal Fermentation Characteristics

The pH of the ruminal fluid from the lambs of both treatment groups was above the threshold pH values of subacute ruminal acidosis (pH > 5.6) [30], indicating that using CV hay instead of AH did not induce acidosis. The pH of the ruminal fluid from lambs fed CVG group was within the optimal pH (6.0 < pH < 6.8) range for fiber digestion [31]. Ruminal NH_3_-N concentration of CVG group was lower than that of the CON group, suggesting that CV hay improved the utilization of ruminal NH_3_-N, indicating a reduction in ruminal protein degradation. The reason for lower NH_3_-N concentration in CVG group could be related to their condensed tannin (CT) concentration (AH vs. CV hay = 1.37 vs. 7.07 mg/g, unpublished), since CT-mediated reductions in the ruminal protein degradation are usually associated with a decline in the concentration of ruminal ammonia [32]. However, despite the negative effect of CV hay on rumen NH_3_-N concentration compared to AH, it did not negatively affect animal productive performance as indicated by the higher growth rate and lower FCR observed in CVG group, probably because CV hay contains higher CT levels and can shift the protein digestion site from the rumen to the small intestine, resulting in improved protein utilization [32].

Regarding the rumen fermentation characteristics, total VFA was numerically higher in lambs offered CVG diet than in those offered the CON diet, although the molar proportion of individual VFA changed. This variation could due to the lower aNDF content, as well as the rapid rumen fermentation and degradability of CV hay compared to AH [33], as the rumen is the main site for fiber digestion [32]. Our results agree with the findings of Huang et al. [6], in which replacing AH with CV hay in the diet of fattening lambs did not affect the total VFA concentration. The significantly higher molar proportion of butyrate isovalerate and lower molar proportion of acetate and valerate in lambs fed CVG diet compared to those fed CON diet, which might be linked to decreased aNDF degradability, lower CH_4_ generation, and high CT content in CV hay. Similar differences were observed in vitro [34] and in vivo [35].

### 4.3. Methane Emission

In this study, CH_4_ emission predicted by the model of Ramin and Huhtanen [29] showed that lambs fed CVG diet had the similar predicted CH_4_ emissions per unit of AFI compared to the control diet. However, lambs fed CVG diet had the lower predicted CH_4_ emissions per unit of ADG. This was a result of the high value for ADG in lambs fed the CVG diet compared to that of lambs fed CON diet. Soltan et al. [36] stated that when comparing CH_4_ emission expressed relative to AFI, it is preferable to determine emission per unit of animal production. Eckard et al. [37] noted that the principal interest of animal producers is the possibility of redirecting energy retained by reduction of CH_4_ emission towards milk or body weight gain. Following this view, CVG diet outperformed the control diet due to the lower predicted CH_4_ emission per unit of body weight gain.

### 4.4. Ruminal Microbial Community Composition

As far as we know, this is the first study reporting the effects of CV hay on the rumen microbiota of lambs. The diversity and richness indices are commonly used to evaluate the stability of an ecosystem [38]. Five samples from each group were used for analysis in the present study, which could a possible limitation. In the present study, the rumen samples from CON and CVG groups could be relatively grouped by beta diversity (Figure 4), indicating that microbial diversity across individuals was affected by the forage included in the TMR, in line with the previous studies that found that the rumen bacterial community was strongly associated with feed type [39]. Furthermore, the diversity and richness of the rumen microbiota of lambs were similar between the two groups, which was in agreement with the work of Liu et al. [13], where replacing AH with oat hay in the diet of lambs had no adverse effects on the alpha diversity of ruminal bacteria. Similarly, these findings could be commonly validated by previous research in ruminants [40,41]. This result indicated that the substitution of CV hay for AH resulted in a similar establishment of rumen microflora.

Ruminal microbiological samples often play a crucial role in assessing the ruminal tract’s health and digestive function [42,43,44], thus influencing animal performance [45]. Regarding the rumen bacterial community, *Bacteroidetes* and *Firmicutes* were the most dominant phyla in the present study (>81.42% relative abundance), which is consistent with previous research results in lambs [46,47]. In the present study, *Bacteroidetes* tended to be higher (21.6%) and *Proteobacteria* (48.4%) tended to be lower in the CVG group than in the CON group. The higher abundance of *Bacteroidetes* observed in the CVG group may be associated with a higher abundance of pectin lyase and a higher degree of starch degradation [48]. The lower abundance of *Proteobacteria* observed in the CVG group, which includes many members of the microaerophiles or facultative anaerobes, could be associated with trace levels of oxygen passing through rumen epithelial cells [49].

According to the results, microbes compete for available resources in the rumen, although without a direct relationship with their relative abundance. In agreement with a previous report, *Prevotella* was the predominant genus within *Bacteroidetes* in the rumen of sheep [50]. This supports the common notion that the phyla *Firmicutes* and *Bacteroidetes* are the core phyla of the rumen microbiome. Using CV hay instead of AH tended to increase the relative abundance of *Prevotella*, which is associated with starch and plant cell polysaccharide degradation, resulting in the production of acetic acid, succinic acid, and propionic acid [51]. In contrast, using CV hay instead of AH had a significant inhibiting effect on fiber hydrolysis bacteria, *Ruminococcus*, suggesting that replacing AH with CV hay affected rumen microbial composition, but not rumen metabolic function, since total rumen VFA content was similar in the two groups. This can be attributed to rumen resilience and redundancy between microbial groups, i.e., the rumen microbiota was able to resist, adapt, and recover in response to different diets. The lower abundance of *Ruminococcus* (<1.5%) may also partly explain this phenomenon. Furthermore, several bacterial genera showed a strong correlation with rumen VFA of the lambs. *Ruminococcus* were positively correlated with the rumen propionic acid in the lambs. These genera potentially play an important part in the producing propionic acid for lambs [52]. In accordance with a previous report [53], *Ruminococcus albus* abundance was positively associated with ruminal NH_3_-N concentration. However, *Ruminococcus* abundance was negatively correlated with ADG in the present study. Further studies of the microbial ecology and metabolism of these bacteria will reveal the mechanistic basis of their growth performance, thereby promoting their potential application as therapeutic agents or additives to promote animal growth.

## 5. Conclusions

Evaluation of hay quality showed that CV hay had a greater in vitro digestibility of DM and NDF than alfalfa hay. Feeding a CVG diet enhanced ADG, reduced predicted CH_4_ emission expressed relative to ADG, and altered rumen fermentation by reducing ruminal NH_3_-N and altering molar proportion of short-chain fatty acids. In addition, lambs offered the CVG diets exhibited a decreased relative abundance of *Ruminococcus* compared with lambs offered CON diet. Overall, common vetch hay has the potential to provide a novel option to lessen dependence on imported alfalfa hay.

## Figures and Tables

**Table 1:** Ingredients (% as-fed basis) and chemical composition of the experimental diets.

Title	Diet ^1^		Common Vetch Hay	Alfalfa Hay
	CON	CVG		
Ingredients				
Alfalfa	40.00	0.00		
Common vetch	0.00	40.00		
Brittle culm rice stover	20.00	20.00		
Corn	28.00	28.00		
Wheat bran	2.91	6.60		
Cottonseed meal	5.40	1.85		
Salt	0.70	0.70		
Calcium carbonate	1.10	1.00		
Calcium bicarbonate	0.89	0.85		
Mineral-vitamin premix ^2^	1.00	1.00		
F:C ratio ^3^	40:60	40:60		
Chemical composition ^4^				
Dry matter, %	89.81	89.52	93.10	92.51
Crude protein, % DM	14.10	14.63	20.82	17.24
Neutral detergent fiber, % DM	35.57	35.47	34.21	36.14
Acid detergent fiber, % DM	23.76	22.06	23.09	27.42
Ash, % DM	8.47	10.97	15.63	9.77
Calcium, % DM	0.90	0.89	0.39	0.28
Phosphorus, % DM	0.50	0.51	0.26	0.25
Metabolizable energy (MJ/kg) ^5^	13.40	13.40		

^1^ CON = diet containing alfalfa hay; CVG = diet containing common vetch hay; DM, dry matter. ^2^ Composition, mg/kg: Fe 1500, Cu 150, Mn 600, Zn 1000, vitamin A 25,000 IU, vitamin D3 40,000 IU, vitamin E 400 mg/kg. ^3^ F:C ratio = proportion between the amounts of roughage and concentrate in the diet. ^4^ Nutrient levels of total mixed ration, common vetch hay and alfalfa hay were measured. ^5^ Estimated as metabolizable energy (MJ/kg) = 3.61 × (88.9 − %Acid detergent fiber × 0.779)/100 × 4.184 [23].

**Table 2:** The parameters of ruminal fermentation of feedlotting lambs fed CON and CVG diets.

Items	Diet ^1^		SEM	*p*-Value
	CON	CVG		
pH	5.86	6.05	0.102	0.406
NH_3_-N (mg/mL)	17.36 ^a^	13.29 ^b^	0.975	0.032
Total VFA (mmol L^−1^)	56.59	61.23	2.77	0.460
Acetate (% molar)	71.73 ^a^	67.45 ^b^	0.839	0.005
Propionate (% molar)	19.44	17.27	0.711	0.158
Butyrate (% molar)	4.66 ^b^	11.38 ^a^	1.10	<0.001
Valerate (% molar)	2.12 ^a^	0.95 ^b^	0.194	<0.001
Isobutyrate (% molar)	1.34	1.26	0.083	0.674
Isovalerate (% molar)	0.71 ^b^	1.69 ^a^	0.212	0.017
Acetate: Propionate	3.75	3.94	0.139	0.547

^a,b^ Values within a row with different lowercase superscript letters differ significantly at *p* < 0.05. ^1^ CON = diet containing alfalfa hay; CVG = diet containing common vetch hay; SEM = standard error of the mean.

**Table 3:** The predicted methane emission of growing Huang-huai lambs fed CON and CVG diets.

Items	Diet ^1^		SEM	*p*-Value
	CON	CVG		
L/day ^2^				
Minimum	55.32	55.16	0.23	0.883
Maximum	88.61	88.41	0.28	0.884
L·kg^−1^·ADG^−1^				
Minimum	343.65 ^a^	265.01 ^b^	16.43	0.015
Maximum	550.60 ^a^	425.08 ^b^	26.42	0.016

^a,b^ Values within a row with different lowercase superscript letters differ significantly at *p* < 0.05. ^1^ ADG = average daily gain; CON = diet containing alfalfa hay; CVG = diet containing common vetch hay; SEM = standard error of the mean. ^2^ Total CH_4_ production (L/day) = CH_4_ (L/day) = 20 × (±12.1) + 35.8 × (±2.87) × average feed intake − 0.50 × (±0.132) × average feed intake [29].

**Table 4:** Alpha diversity indices analysis of operational taxonomic units (OTUs) of the ruminal microbiota of sheep fed CON and CVG diets.

Items	Diet ^1^		SEM	*p*-Value
	CON	CVG		
OTUs	1057	1071	16.77	0.926
Chao1	1353.62	1372.55	14.22	0.912
Simpson’s index	0.95	0.87	0.009	0.317
Shannon index	6.40	5.44	0.175	0.278
Goods_coverage (%)	99.34	99.28	0.013	0.503

^1^ CON = diet containing alfalfa hay; CVG = diet containing common vetch hay; SEM = standard error of the mean.

## Data Availability

Data used and analyzed during this study are available from the corresponding author on reasonable request.

## Supplementary Materials

The following supporting information can be downloaded at: https://www.mdpi.com/article/10.3390/ani14152182/s1, Table S1: Microbial community analysis at the phylum level (relative abundance > 1%) of microbiome obtained from the rumen contents of lambs fed fed CON and CVG diet. Table S2: Microbial community analysis at the genera level (top 15 genera) of microbiome obtained from the rumen contents of lambs fed CON and CVG diet.
