# Supplementary material for: Effect of Replacing Alfalfa Hay with Common Vetch Hay in Sheep Diets on Growth Performance, Rumen Fermentation and Rumen Microbiota
Source: Animals (Basel). 2024 Jul 26;14(15):2182. doi: 10.3390/ani14152182 (PMC11310988; doi:10.3390/ani14152182)
Supplement: Supplementary file 1 [file animals-14-02182-s001.zip › animals-3066469-supplementary.pdf]

**Table S1.** Microbial community analysis at the phylum level (relative abundance >1%) of microbiome obtained from the rumen contents of lambs fed fed CON and CVG diet.

| Item | Diet [1] | | | SEM | *P*-value |
|---|---|---|---|---|---|
| | CON | CVG | Mean | | |
| *Bacteroidetes* | 53.42 | 64.95 | 59.18 | 3.56 | 0.107 |
| *Firmicutes* | 28.00 | 21.01 | 24.50 | 2.84 | 0.267 |
| *Actinobacteria* | 7.31 | 3.77 | 5.54 | 2.97 | 0.502 |
| *Proteobacteria* | 2.96 | 1.29 | 2.13 | 0.505 | 0.076 |
| *Fibrobacteres* | 0.96 | 3.03 | 2.00 | 0.286 | 0.233 |
| *Tenericutes* | 2.08 | 2.08 | 1.91 | 0.392 | 0.754 |
| *Spirochaetes* | 2.01 | 1.64 | 1.82 | 0.509 | 0.692 |

[1] CON= diet containing alfalfa hay; CVG = diet containing common vetch hay.

**Table S2.** Microbial community analysis at the genera level (top 15 genera) of microbiome obtained from the rumen contents of lambs fed fed CON and CVG diet.

| Item | Diet [1] | | | SEM | *P*-value |
|---|---|---|---|---|---|
| | CON | CVG | Mean | | |
| *Prevotella* | 22.65 | 41.67 | 32.16 | 5.45 | 0.095 |
| *Succiniclasticum* | 1.56 | 3.57 | 2.57 | 0.710 | 0.095 |
| *RFN20* | 3.21 | 1.56 | 2.38 | 0.966 | 0.690 |
| *Fibrobacter* | 0.96 | 3.03 | 2.00 | 0.788 | 0.841 |
| *Butyrivibrio* | 1.56 | 2.03 | 1.80 | 0.270 | 0.548 |
| *Treponema* | 1.93 | 1.57 | 1.75 | 0.40 | 1.000 |
| *Bifidobacterium* | 0.02 | 2.03 | 1.02 | 0.918 | 0.151 |
| *Ruminococcus* | 1.42 [a] | 0.53 [b] | 0.97 | 0.180 | 0.032 |
| *Oscillospira* | 1.16 | 0.69 | 0.93 | 0.289 | 0.421 |
| *Selenomonas* | 1.58 | 0.26 | 0.92 | 0.488 | 0.222 |
| *CF231* | 0.94 | 0.86 | 0.90 | 0.227 | 0.690 |
| *Desulfobulbus* | 1.15 | 0.34 | 0.75 | 0.250 | 0.095 |
| *Pseudobutyrivibrio* | 0.47 | 0.69 | 0.58 | 0.140 | 0.690 |
| *Desulfovibrio* | 0.94 | 0.15 | 0.54 | 0.207 | 0.151 |
| *Adlercreutzia* | 0.72 | 0.23 | 0.47 | 0.160 | 0.095 |

[a,b] Values within a row with different lowercase superscript letters differ significantly at $P < 0.05$.

[1] CON= diet containing alfalfa hay; CVG = diet containing common vetch hay.
